# Phase‐Programmed Microneedles Enable Sequential Hydrogen and Quercetin Release for Scar‐Reduced Regeneration of Burn Wounds

**DOI:** 10.1002/advs.78512

**Published:** 2026-10-28

**Authors:** Yongnian Zeng, Zhigang Chu, Huiyuan Yang, Wen Zhang, Peng Jiang, Jiapeng Lei, Siyu Zhang, Qiongfang Ruan, Shuhua Liu, Wei Li

**Affiliations:** ^1^ Department of Burns, Tongren Hospital of Wuhan University (Wuhan Third Hospital) School of Pharmaceutical Sciences Wuhan University Wuhan People's Republic of China; ^2^ Key Laboratory of Advanced Pharmaceutics School of Health China Three Gorges University Yichang People's Republic of China; ^3^ Taikang Center for Life and Medical Sciences Wuhan University Wuhan People's Republic of China

**Keywords:** burn wounds, hydrogen, microneedles, scar‐reduced healing, sustained release

## Abstract

Burn wounds complicated by bacterial infection and fibrotic scarring remain a major clinical challenge, demanding therapeutics capable of coordinated infection control, inflammation modulation, and tissue regeneration. Here, we report a phase‐programmed microneedle (MN) patch (QR/AB‐MNs) that enables spatiotemporally controlled release of ammonia borane (AB) and quercetin (QR) to achieve synergistic antimicrobial and anti‐fibrotic therapy. The MNs comprise a poly (lactic‐co‐glycolic acid) (PLGA) shell encapsulating QR and a sulfobutylether‐β‐cyclodextrin (SBECD) core loaded with the H_2_‐releasing prodrug AB. Upon exposure to the mildly acidic wound microenvironment, the system triggers sustained generation of hydrogen (H_2_) and AB degradation products, effectively eliminating bacteria and biofilms while mitigating oxidative stress. Subsequently, gradual PLGA degradation ensures prolonged QR release, suppressing fibroblast hyperproliferation and TGF‐*β* mediated collagen deposition, thereby preventing hypertrophic scarring. In murine and rabbit burn models, QR/AB‐MNs markedly accelerated wound closure, reduced inflammation and bacterial burden, restored skin appendages, and yielded scar‐reduced regeneration with native‐like collagen architecture. Transcriptomic profiling further revealed that QR/AB‐MNs orchestrate a broad anti‐fibrotic transcriptional program, concurrently attenuating pro‐fibrotic signaling, cell‐extracellular matrix interactions, and collagen synthesis. Collectively, this programmable MN platform integrates redox modulation, anti‐infective therapy, and matrix remodeling to achieve comprehensive, scarless‐like healing of infected burn wounds.

## Graphical abstract

A phase‐programmed microneedle patch, comprising a poly (lactic‐co‐glycolic acid) (PLGA) shell and a sulfobutylether‐β‐cyclodextrin (SBECD) core, is designed for sequential hydrogen and quercetin release for scar‐reduced regeneration of burn wounds.

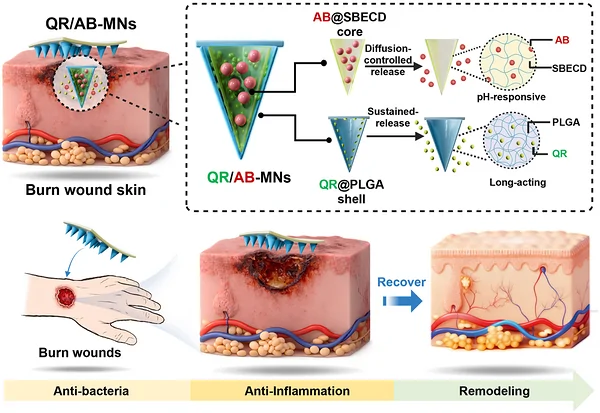

## 1 Introduction

Burn injuries represent the fourth most common type of traumatic injury worldwide and remain a major clinical and socioeconomic burden [1, 2]. Typically, burn wounds arise from exposure to heat, radiation, electricity, or chemicals. Depending on tissue damage depth, burns are classified as first‐, second‐, or third‐degree, with the latter two often necessitating surgical intervention [3, 4]. Owing to severe skin barrier disruption and impaired immune function, burn wounds are highly susceptible to microbial infections‐particularly by *Pseudomonas aeruginosa* (*P. aeruginosa*)‐before full epithelialization [1, 5, 6]. Bacterial colonization and infection impede critical phases of wound healing, including inflammation resolution, re‐epithelialization, and collagen remodeling, and may even progress to sepsis in severe cases [7]. Moreover, excessive inflammatory responses and aberrant fibroblast activation frequently result in hypertrophic scarring, further complicating recovery [8]. Recently, a range of advanced wound dressings, including reactive oxygen species (ROS)‐responsive hydrogels [9, 10], nitric oxide (NO)‐releasing nanofiber sheets [11], and electrospun wound dressings [12, 13], have shown considerable potential for regulating oxidative stress, controlling infection, and promoting tissue regeneration. However, the therapeutic effects of many of these surface‐applied dressings are largely confined to the wound interface, which may limit drug penetration into deeper viable tissues, particularly in the presence of burn eschar. Moreover, most existing dressings are primarily designed to promote wound closure or control infection and do not simultaneously address post‐burn hypertrophic scar formation [14]. Thus, designing a multifunctional therapeutic platform that coordinates infection control, tissue regeneration, and scar inhibition remains an unmet challenge.

Burn wound healing is a highly orchestrated process involving distinct yet overlapping phases of inflammation, proliferation, and remodeling [15, 16]. During the inflammatory phase, immune cells secrete cytokines (e.g., interleukins and TNF‐*α*) to combat infection, while in the proliferative phase, fibroblasts, endothelial cells, and keratinocytes promote tissue repair through migration, proliferation, and extracellular matrix deposition. Recently, hydrogen (H_2_)‐based therapies have attracted considerable attention because of the unique physicochemical and biological properties of H_2_ [17, 18, 19, 20], particularly its potential to regulate burn wound healing by alleviating oxidative stress and accelerating wound closure [21, 22, 23]. However, current H_2_ delivery strategies, including inhalation therapy, H_2_‐rich water, and H_2_‐loaded hydrogels, are limited by low solubility, transient bioavailability, and poor tissue penetration, thereby constraining their clinical translation [24]. Furthermore, excessive fibroblast proliferation and collagen accumulation during the remodeling phase can induce hypertrophic scarring. Quercetin (QR), a naturally occurring flavonoid, has demonstrated strong anti‐fibrotic and anti‐scarring effects through inhibition of TGF‐*β* signaling and collagen I synthesis, thereby mitigating pathological scar development [25, 26, 27]. Nevertheless, the therapeutic efficacy of QR is often compromised by limited tissue penetration and the need for repeated administration throughout the prolonged wound‐healing process. Therefore, the development of a long‐acting, phase‐responsive therapeutic platform capable of sustained H_2_ delivery while simultaneously preventing fibrotic scarring represents an urgent unmet need in burn wound management.

Microneedle (MN) technology has gained prominence as a minimally invasive transdermal delivery platform for various medical applications, including cancer [28, 29, 30], diabetes [31, 32, 33], obesity [34, 35], and infections [36, 37, 38]. Antimicrobial MNs, in particular, exhibit remarkable potential in promoting wound healing by disrupting bacterial biofilms and enhancing local drug delivery [39, 40]. Although staged‐release MNs have been explored for sequential drug delivery, most existing systems are designed primarily to achieve temporal separation of drug exposure rather than to coordinate therapeutically distinct functions with the dynamic biology of wound repair [41, 42]. In infected burn wounds, this distinction is particularly important because early infection control and tissue regeneration must be followed by suppression of excessive fibroblast activation and pathological matrix remodeling [43, 44]. A platform that continuously promotes proliferation throughout the healing process may therefore be counterproductive during the remodeling stage. Accordingly, an effective strategy should not only control when therapeutic agents are released but also assign distinct biological functions to different stages of healing.

To this end, we developed a phase‐programmed core–shell MN system (QR/AB‐MNs) capable of sequentially regulating the inflammatory, proliferative, and remodeling phases to achieve scar‐reduced healing of infected burn wounds. As illustrated in Figure 1, the QR/AB‐MNs integrate two bioactive modules: a water‐soluble sulfobutylether‐β‐cyclodextrin (SBECD) core loaded with the H_2_‐releasing prodrug ammonia borane (AB), and a biodegradable poly (lactic‐co‐glycolic acid) (PLGA) shell encapsulating QR. Upon application to infected burn sites, the AB core is activated by the mildly acidic wound microenvironment to continuously release H_2_ and boric acid, which exert potent antibacterial and antioxidative effects while promoting keratinocyte re‐epithelialization, fibroblast migration, and macrophage polarization. Subsequently, gradual PLGA shell degradation enables sustained QR release over 30 days, effectively attenuating fibroblast hyperplasia, modulating collagen I/III balance, and preventing hypertrophic scarring. Thus, rather than simply co‐delivering two therapeutic agents, QR/AB‐MNs spatially and temporally coordinate regenerative and anti‐fibrotic functions within a single minimally invasive platform, thereby matching therapeutic activity to the changing biological requirements of infected burn wounds (Table S1).

**FIGURE 1:**
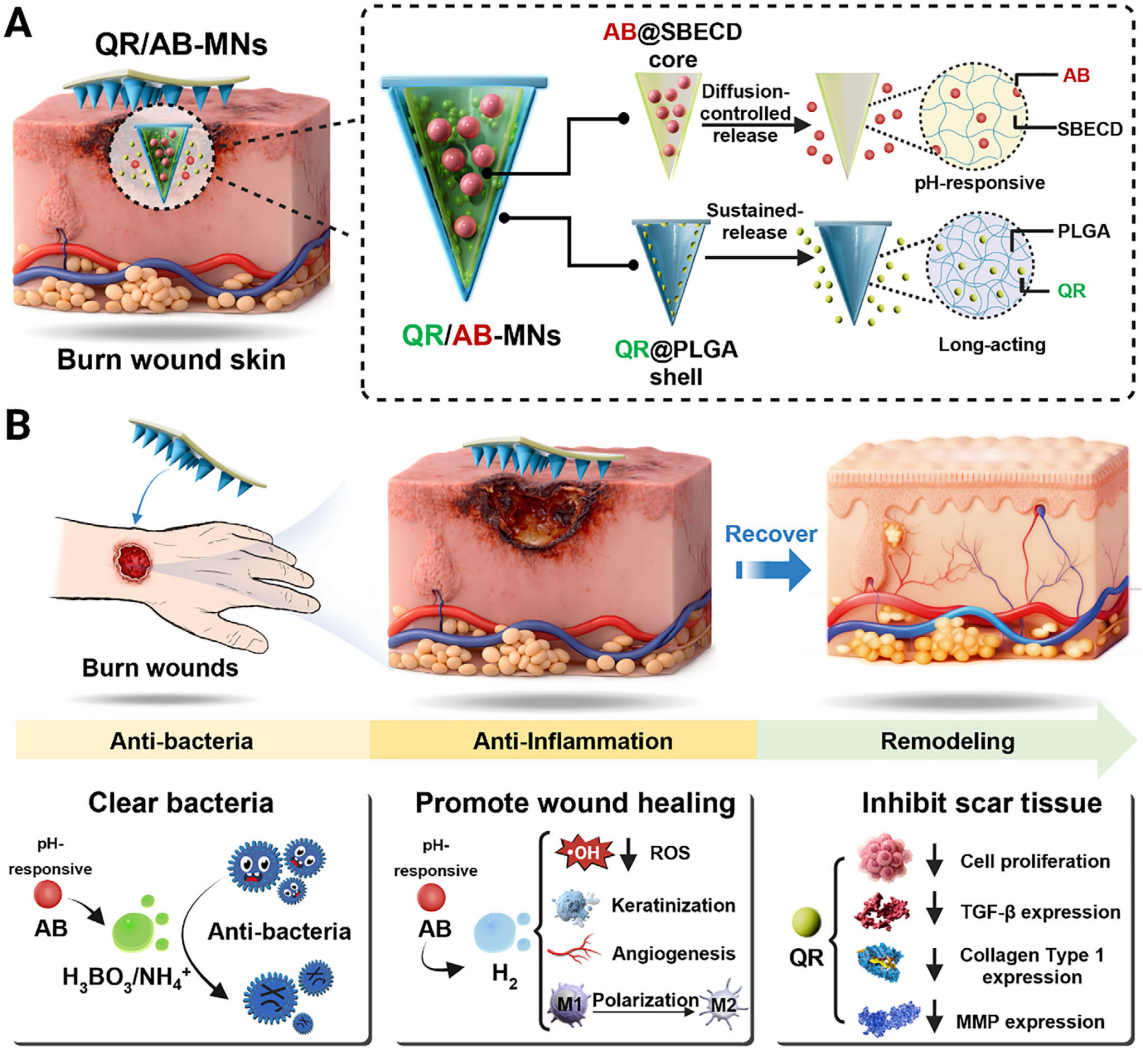
Schematic illustration of the phase‐programmed core–shell MNs (QR/AB‐MNs) enabling scarless regeneration of infected burn wounds through sequential H_2_ release and QR delivery. (A) Structural design and composition of the QR/AB‐MNs. (B) Schematic illustration of the working mechanism of QR/AB‐MNs for promoting scarless healing of burn wounds.

## 2 Results and Discussion

### 2.1 Fabrication and Characterization of the QR/AB‐MN Patches

The core–shell structured MN patch (QR/AB‐MNs) for the synergistic treatment of infected burn wounds was fabricated using a micromolding technique (Figure S1A). The MNs consist of a PLGA shell, an SBECD core, and a PLGA “Lid” structure (Figure S1B). A soluble baseplate was formed via vacuum casting to ensure intact implantation of the core–shell structure following skin penetration. The PLGA shell was loaded with the anti‐scarring agent QR to achieve sustained release within the wound bed, while the SBECD core encapsulated the H_2_‐releasing prodrug AB, designed for triggered, sustained hydrogen generation upon exposure to the acidic infected wound microenvironment. Experimental results (Figure 2A and Figure S2) demonstrated that the PLGA lid effectively prevented premature leakage of core contents, maintaining stable core fluorescence intensity over 24 h, in contrast to rapid diffusion observed in lid‐free MNs. Scanning electron microscopy (SEM) characterization revealed arrays of sharply conical MNs with a height of 850 µm, base diameter of 300 µm, and tip diameter of 10 µm (Figure 2B). Moreover, elemental mapping and energy‐dispersive x‐ray spectroscopy (EDS) confirmed the distinct core–shell architecture and precise drug compartmentalization (Figure 2F and Figure S3A), with a measured shell thickness of 31.9 ± 1.4 µm (Figure 2E).

**FIGURE 2:**
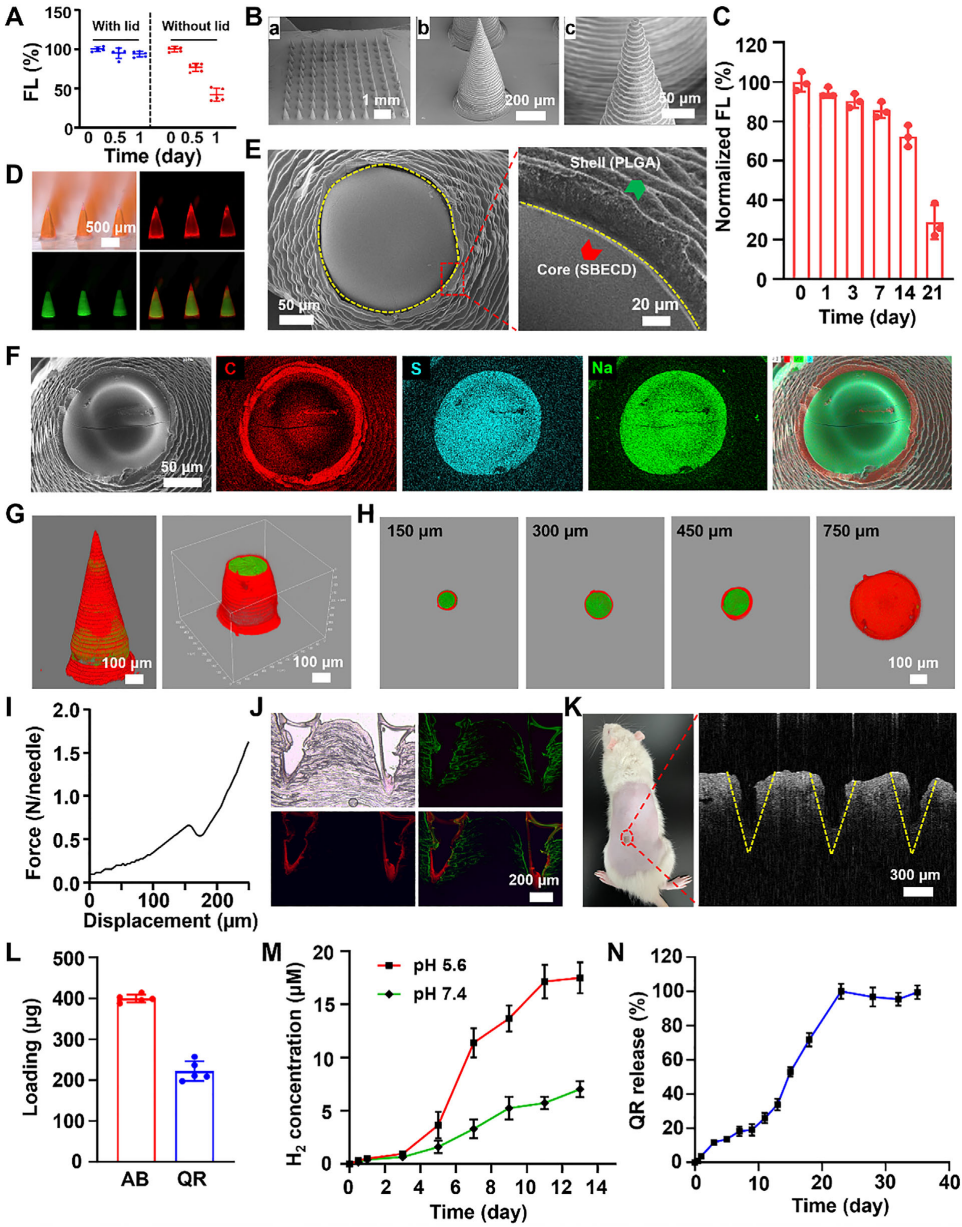
Characterization of QR/AB‐MNs. (A) Quantitative analysis of fluorescence intensity in the core region of MNs with or without lid structures across various time intervals (*n* = 5). (B) SEM images of a QR/AB‐MN patch (a), an individual MN (b), and the MN tip part (c). (C) Statistical analysis of fluorescence intensity changes over time in the core of core–shell MNs with lid (*n* = 3). (D) Representative illustration of the core–shell architecture in QR/AB‐MNs, featuring a Nile red‐labeled PLGA shell (red) and rhodamine green‐labeled SBECD core (green). (E) Cross‐sectional SEM image of a single MN (left) and its magnified view (right), with green and red arrows indicating the core and shell layer, respectively. (F) Elemental mapping of the cross‐section of the shell and core of QR/AB‐MNs. (G) Representative confocal laser scanning microscopy images of intact and truncated QR/AB‐MNs with Nile red‐labeled PLGA shell (red) and rhodamine green‐labeled SBECD core (green). (H) Representative cross‐sectional confocal microscopy images of core–shell MNs at different depths. (I) Force‐displacement curve of QR/AB‐MNs. (J) Frozen section images of porcine skin after the MNs insertion ex vivo. (K) Optical coherence tomography (OCT) of rat skin following MN patch insertion. (L) Statistical chart of the drug loading capacity of AB and QR in the QR/AB‐MN patch (*n* = 5). (M) Hydrogen (H_2_) generation profile of QR/AB‐MNs under simulated physiological conditions (*n* = 5). (N) In vitro cumulative release kinetics of QR from MNs (*n* = 5). Data are presented as mean ± SD.

Stereo fluorescence microscopy (Figure 2D and Figure S3B) and Confocal laser scanning microscopy (CLSM) (Figure 2G) further validated the stratified core–shell structure, revealing a sharp, non‐interpenetrating interface between the PLGA shell and SBECD core layers. Cross‐sectional imaging at various depths demonstrated the complete encapsulation of the core by the PLGA lid (Figure 2H). Mechanical testing demonstrated robust structural integrity, with each MN sustaining a force of 0.65 N (Figure 2I), which is sufficient for effective transdermal insertion. Ex vivo penetration experiments using porcine skin (Figure 2J) and optical coherence tomography (OCT) imaging (Figure 2K) further validated successful skin penetration and efficient dual‐drug delivery across the epidermal barrier. To further assess the translational feasibility of MN application to burn wounds, ex vivo penetration studies were performed using porcine skin tissue containing burn eschar. H&E staining revealed distinct MN‐generated microchannels traversing the eschar and extending into the underlying viable dermis, confirming that the MNs can penetrate the eschar barrier and establish delivery pathways to viable tissue (Figure S4).

Drug loading per patch was 399.4 ± 8.7 µg for AB and 222.1 ± 21.7 µg for QR (Figure 2L and Figure S5A,B). In vitro release studies (Figure S5C and Figure 2M,N) and simulated release modeling (Figure S5D and Figure 2C) demonstrated sustained release profiles: the core enabled sustained H_2_ generation under acidic pH stimulation, with the dissolved H_2_ concentration gradually increasing to a maximum of 17.5 ± 1.5 µm on day 13, while the release of QR from the PLGA shell persisted for over 30 days. SEM imaging monitored the morphological evolution of the PLGA shell in simulated wound exudate (Figure S6). The degradation profile of the PLGA shell mirrored its drug release kinetics, maintaining structural integrity for over 25 days. Significant pore formation appeared around day 10, potentially attributable to gas generation from the core AB. Furthermore, erythrocyte hemolysis assays (Figure S7A,B) and live/dead cell staining (Figure S7C) confirmed favorable biocompatibility of the core–shell MN patches.

### 2.2 Antimicrobial Properties of QR/AB‐MN Patches In Vitro

Degradation products generated from AB hydrolysis exhibit potent antimicrobial activity [45, 46]. To evaluate its efficacy, the minimum inhibitory concentrations (MIC) of free AB were first determined. AB exhibited MIC values of 31.25 µg/mL against *Escherichia coli* (*E. coli*), 62.5 µg/mL against *Pseudomonas aeruginosa* (*P. aeruginosa*), and 31.25 µg/mL against *Staphylococcus aureus* (*S. aureus*) (Figure S8A and Figure 3A). To further elucidate the contribution of H_2_ to the antibacterial activity of AB, the antibacterial effects of freshly prepared AB and aged AB solution, in which the generated H_2_ had largely dissipated, were compared. The aged AB solution exhibited antibacterial activity comparable to that of freshly prepared AB, indicating that H_2_ does not make a detectable contribution to the direct antibacterial activity under our experimental conditions. Instead, the antibacterial effect is primarily associated with the non‐gaseous degradation products generated during AB hydrolysis (Figure S9). The antimicrobial activity of QR/AB‐MNs was subsequently assessed by colony‐forming unit (CFU) counting. Solid culture media treated with AB‐containing MNs displayed negligible bacterial growth (Figure S8B). Consistently, live/dead bacterial staining showed that treatment with AB‐MNs reduced bacterial viability of *P. aeruginosa* to below 5% (Figure 3D,E), demonstrating the excellent antibacterial efficacy of QR/AB‐MNs. SEM imaging further corroborated these findings. Bacteria treated with AB‐free MNs retained smooth, intact, and turgid morphologies (Figure 3B), whereas those exposed to QR/AB‐MNs appeared shrunken and roughened, exhibiting severe membrane disruption, cytoplasmic leakage, and structural collapse. These observations suggest that QR/AB‐MNs exert bactericidal effects primarily through bacterial membrane destruction. Furthermore, similar antibacterial activity was observed against *E. coli* and *S. aureus* (Figure S10). Collectively, these results confirm that AB incorporation endows the MNs with potent and broad‐spectrum antimicrobial activity.

**FIGURE 3:**
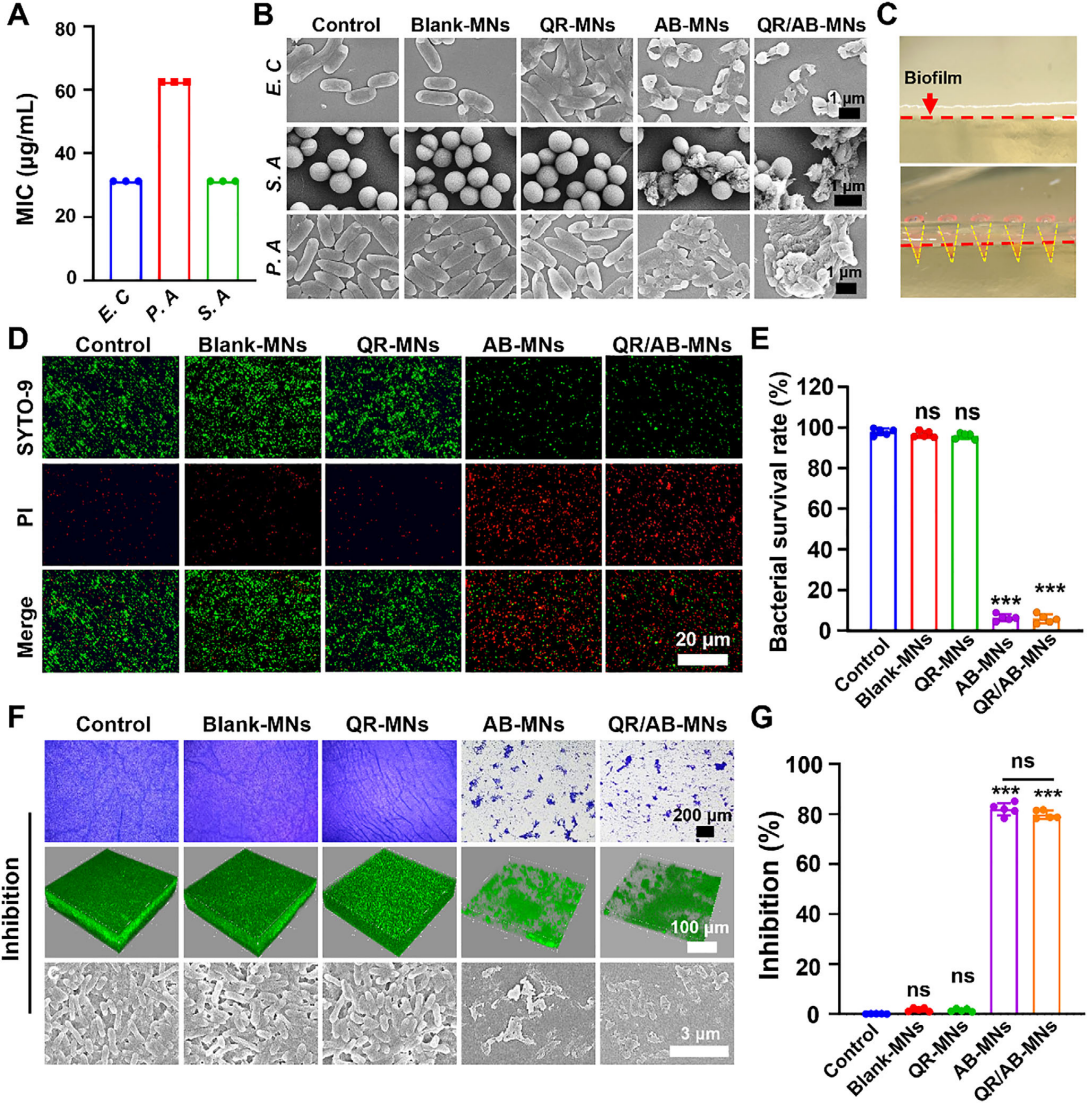
Evaluation of the antibacterial properties of QR/AB‐MNs. (A) Minimum Inhibitory Concentration (MIC) values of AB against *E. coli*, *S. aureus*, and *P. aeruginosa* (*n* = 3). (B) Representative SEM micrographs of *E. coli*, *S. aureus*, and *P. aeruginosa* following treatment with different MNs. (C) Representative image demonstrating MNs penetration through bacterial biofilm; the red dashed line indicates the biofilm boundary. (D) Live/dead bacterial viability assay of *P. aeruginosa* after treatment with various MNs groups. Green fluorescence indicates live bacteria; red indicates dead bacteria. (E) Quantitative analysis of bacterial survival rates across different experimental groups (*n* = 5). (F) Inhibitory effects of MNs on nascent biofilm formation. (top) Photographs of crystal violet‐stained nascent biofilms, (middle) confocal 3D reconstructions of SYTO‐9‐stained biofilms post‐treatment, and (bottom) SEM images of gradient‐dehydrated nascent biofilms after different treatments. (G) Statistical analysis of biofilm inhibition rates across treatment groups (*n* = 5). Data are presented as mean ± SD. ^***^ *p* < 0.001, ns indicates no significance.

Bacterial biofilms represent a major barrier in chronic wound treatment due to their dense extracellular polymeric substance (EPS) matrix, which limits antimicrobial penetration and efficacy [47, 48]. The anti‐biofilm performance of the core–shell QR/AB‐MNs was systematically investigated against *P. aeruginosa* biofilms. As illustrated in Figure 3C, the MNs effectively penetrated biofilms and delivered antimicrobial agents beneath the EPS layer to disrupt the biofilm structure. Anti‐biofilm assays revealed that QR/AB‐MNs markedly inhibited biofilm formation. Crystal violet staining, CLSM 3D reconstruction, and SEM imaging demonstrated reduced biofilm thickness, loosened architecture, and decreased bacterial density following treatment (Figure 3F). Quantitative analysis showed up to 80% inhibition of nascent biofilm formation (Figure 3G). Moreover, QR/AB‐MNs exhibited significant efficacy against mature biofilms, achieving eradication rates of up to 60% (Figure S8C,D). Together, these multi‐modal characterizations confirm that QR/AB‐MNs can effectively penetrate bacterial biofilms and release active antimicrobial payloads, enabling highly efficient anti‐biofilm therapy.

### 2.3 Wound Healing Assessment In Vitro

Excessive ROS accumulation in burn wounds is a key pathological factor that impairs cellular function and delays healing [49, 50, 51]. In an in vitro skin cell model induced by hydrogen peroxide (H_2_O_2_), a marked reduction in green fluorescence intensity was observed in groups treated with AB‐containing MNs (AB‐MNs and QR/AB‐MNs) (Figure 4A), confirming that QR/AB‐MNs effectively reduced intracellular ROS levels through sustained H_2_ release. Wound healing involves coordinated actions of multiple cell types, including keratinocyte re‐epithelialization and migration, fibroblast proliferation and migration, and vascular endothelial cell reconstruction. In the HaCaT scratch assay, treatment with QR/AB‐MNs achieved up to 60% wound closure within 24 h (Figure S11A,B). Live‐cell time‐lapse imaging further revealed a 178.3% increase in colony area expansion rate compared to the control group (Figure 4B,C), suggesting that the released H_2_ markedly accelerates keratinocyte re‐epithelialization. Similarly, in fibroblast monolayers, QR/AB‐MNs induced a scratch closure rate of 96.8% ± 0.6%, significantly higher than that of other groups (Figure 4D,E). Consistent with these findings, Transwell migration assays demonstrated significantly enhanced fibroblast motility following QR/AB‐MNs treatment (Figure S11C,D). Revascularization plays an essential role in supplying oxygen and nutrients during tissue regeneration. A Matrigel‐based angiogenesis assay showed that both AB‐MNs and QR/AB‐MNs, capable of sustained H_2_ generation, significantly increased tubule length and branching density compared to controls (Figure 4F). Collectively, these results indicate that QR/AB‐MNs activate the proliferative and migratory potential of keratinocytes, fibroblasts, and endothelial cells, thereby promoting multiple key processes involved in wound repair.

**FIGURE 4:**
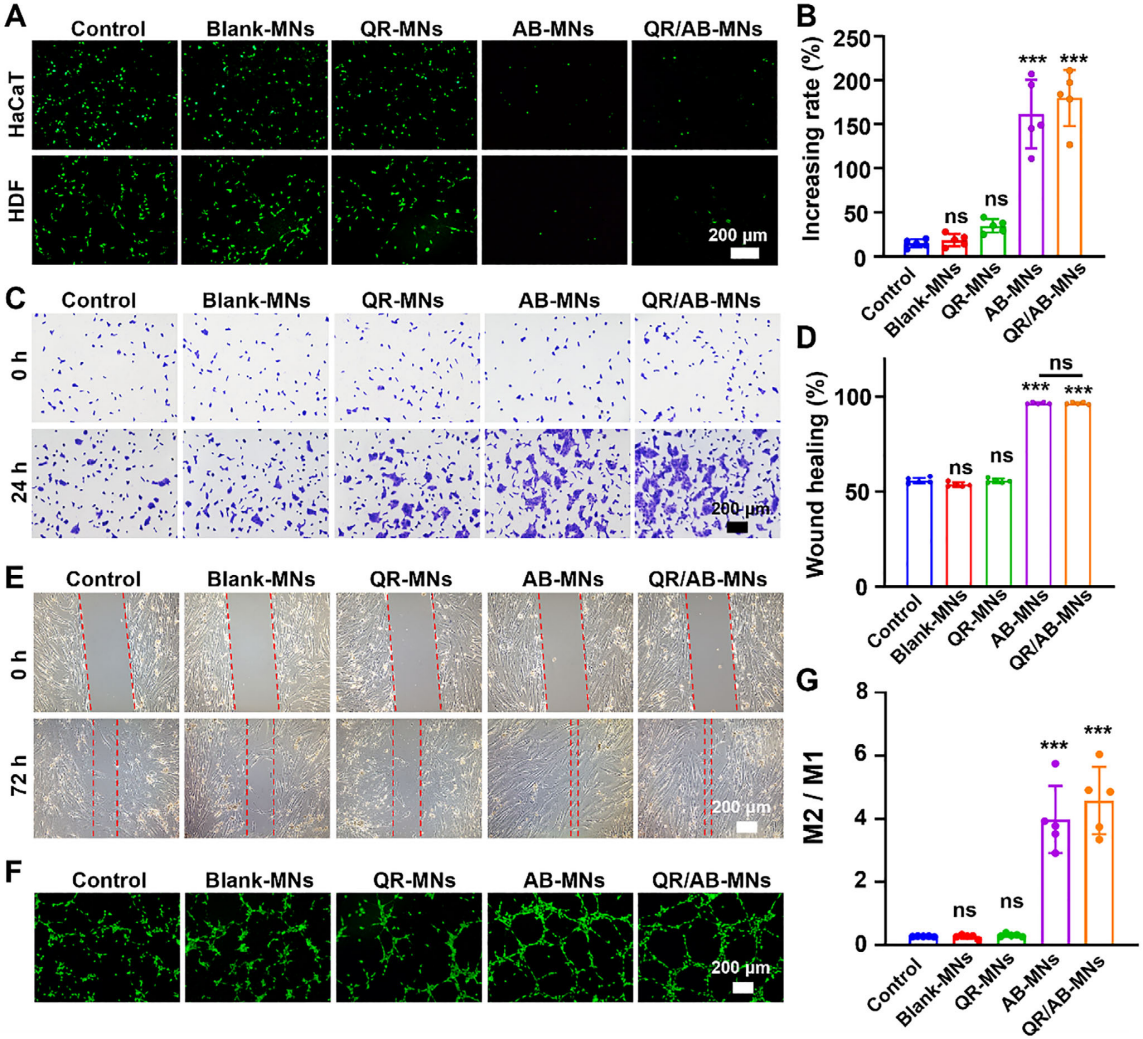
Evaluation of cell migration, epithelialization, angiogenesis, and macrophage polarization following QR/AB‐MNs treatment. (A) Representative fluorescence images of intracellular probe intensity in H_2_O_2_‐induced HaCaT cells and HDF cells. (B) Quantitative analysis of the expansion rate in total epithelialized colony area of HaCaT cells (*n* = 5). (C) Representative images illustrating epithelialization of HaCaT cells across different treatment groups. (D) Quantitative assessment of scratch closure rate in HDF cells (*n* = 5). (E) Representative images of scratch wound closure in HDF cells under various treatments. (F) Confocal microscopy images of tube‐like structures formed by HUVEC cells co‐cultured with different types of MNs. (G) Statistical analysis of the percentage of anti‐inflammatory (M2) and pro‐inflammatory (M1) macrophages in RAW 264.7 cells after co‐culture with different MNs (*n* = 5). Data are presented as mean ± SD. ^***^ *p* < 0.001, ns indicates no significance.

Beyond its antioxidant capacity, molecular H_2_ also modulates inflammatory responses by influencing macrophage polarization. Flow cytometric analysis demonstrated that treatment with AB‐containing MNs significantly elevated the ratio of anti‐inflammatory (M2) to pro‐inflammatory (M1) macrophages, whereas AB‐free MNs maintained a predominantly M1 phenotype with minimal M2 conversion (Figure 4G and Figure S12). These findings highlight the potent anti‐inflammatory properties of QR/AB‐MNs and their capacity to promote a regenerative immune microenvironment conducive to wound healing.

### 2.4 Suppression of Scar Formation In Vitro

QR, a polyhydroxylated flavonoid, has been reported to effectively inhibit hypertrophic scar formation [27]. Notably, QR selectively inhibited the proliferation of human keloid fibroblasts (HKF) while exerting no significant effect on the proliferation of normal human dermal fibroblasts (HDF) under high‐ROS conditions, demonstrating its selective anti‐fibrotic activity and favorable cytocompatibility (Figure S13). To evaluate the anti‐fibrotic potential of QR/AB‐MNs, HKF were employed as a representative model. CCK‐8 assays demonstrated that treatment with QR‐containing MNs (QR‐MNs and QR/AB‐MNs) markedly reduced HKF viability to approximately 50%, in contrast to > 98% viability observed in the control group (Figure 5A), indicating a significant cytostatic effect. Consistently, scratch wound assays revealed substantially larger residual wound gaps after 48 h in the QR‐loaded MN groups (Figure 5B), reflecting impaired fibroblast proliferation and migration. EdU incorporation analysis further confirmed a pronounced reduction in DNA synthesis, with EdU‐positive rates of 17.1% and 18.3% in the QR‐MNs and QR/AB‐MNs groups, respectively, representing an ∼ 80% decrease relative to controls (Figure 5C,D). Similarly, colony formation assays showed a marked decline in clonogenic potential following treatment with QR‐loaded MNs (Figure 5E,F). Collagen gel contraction assays corroborated these findings (Figure 5G), revealing significant attenuation of fibroblast‐mediated matrix contraction in both groups.

**FIGURE 5:**
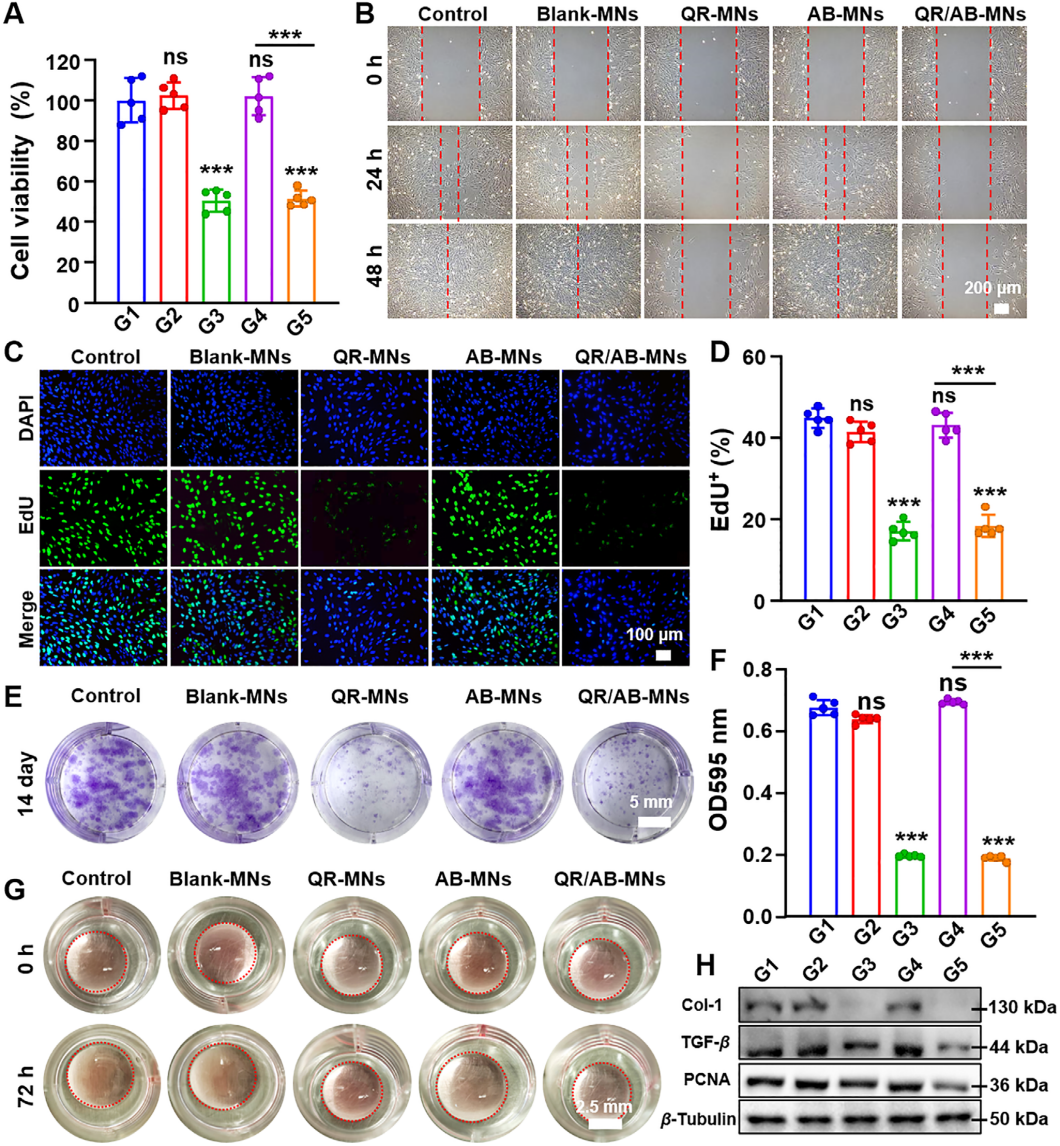
Evaluation of the effects of different MNs on human keloid fibroblast (HKF) behavior. (A) Cell viability of HKFs co‐cultured with different MNs. (B) Representative images of scratch wound assays showing HKF migration after treatment. (C) Immunofluorescence images of EdU incorporation in HKF cells. (D) Quantitative analysis of EdU‐positive cell ratios across groups. (E) Representative images of colony formation assays. (F) Quantitative assessment of colony formation efficiency. (G) Collagen gel contraction assays reflecting fibroblast contractile activity. (H) Western blot analysis of collagen 1 (Col‐1), TGF‐*β*, and PCNA expression in HKFs treated with different MNs. Data are represented as mean ± SD (*n* = 5). ***P < 0.001, ns indicates no significance. (G1: Control, G2: Blank‐MNs, G3: QR‐MNs, G4: AB‐MNs, G5: QR/AB‐MNs).

Mechanistic analyses suggested that QR/AB‐MNs exert anti‐fibrotic effects through coordinated suppression of multiple scar‐associated signaling pathways. Western blot analysis indicated downregulation of collagen I (Col‐I) and proliferating cell nuclear antigen (PCNA), accompanied by reduced activation of the TGF‐*β* signaling cascade (Figure 5H and Figure S14). To elucidate the transcriptional mechanisms underlying these effects, transcriptomic profiling of HKF treated with QR/AB‐MNs was performed. The results demonstrated significant downregulation of key pro‐fibrotic genes, including collagen genes (Col1‐*α*1, Col1‐*α*2), the key fibrogenic regulator TGF‐*β*1, and matrix‐remodeling enzymes (MMP2, MMP11) (Figure 6A,B). Gene set enrichment analysis (GSEA) further revealed coordinated suppression of fibrosis‐related pathways, particularly those involved in extracellular matrix (ECM)‐receptor interaction and focal adhesion, suggesting impaired ECM sensing and contractility in treated fibroblasts (Figure 6C,D). Kyoto Encyclopedia of Genes and Genomes (KEGG) and Gene Ontology (GO) term analysis corroborated these findings, identifying enrichment in biological processes related to the regulation of cell migration and ECM organization, with corresponding cellular component and molecular function categories localizing to ECM structures and binding interactions (Figure 6E,F). Collectively, these genomic findings support the involvement of a broad anti‐fibrotic transcriptional program, simultaneously attenuating pro‐fibrotic signaling, cell‐ECM interactions, and collagen deposition, thereby providing a molecular foundation for its programmatic scar‐prevention efficacy.

**FIGURE 6:**
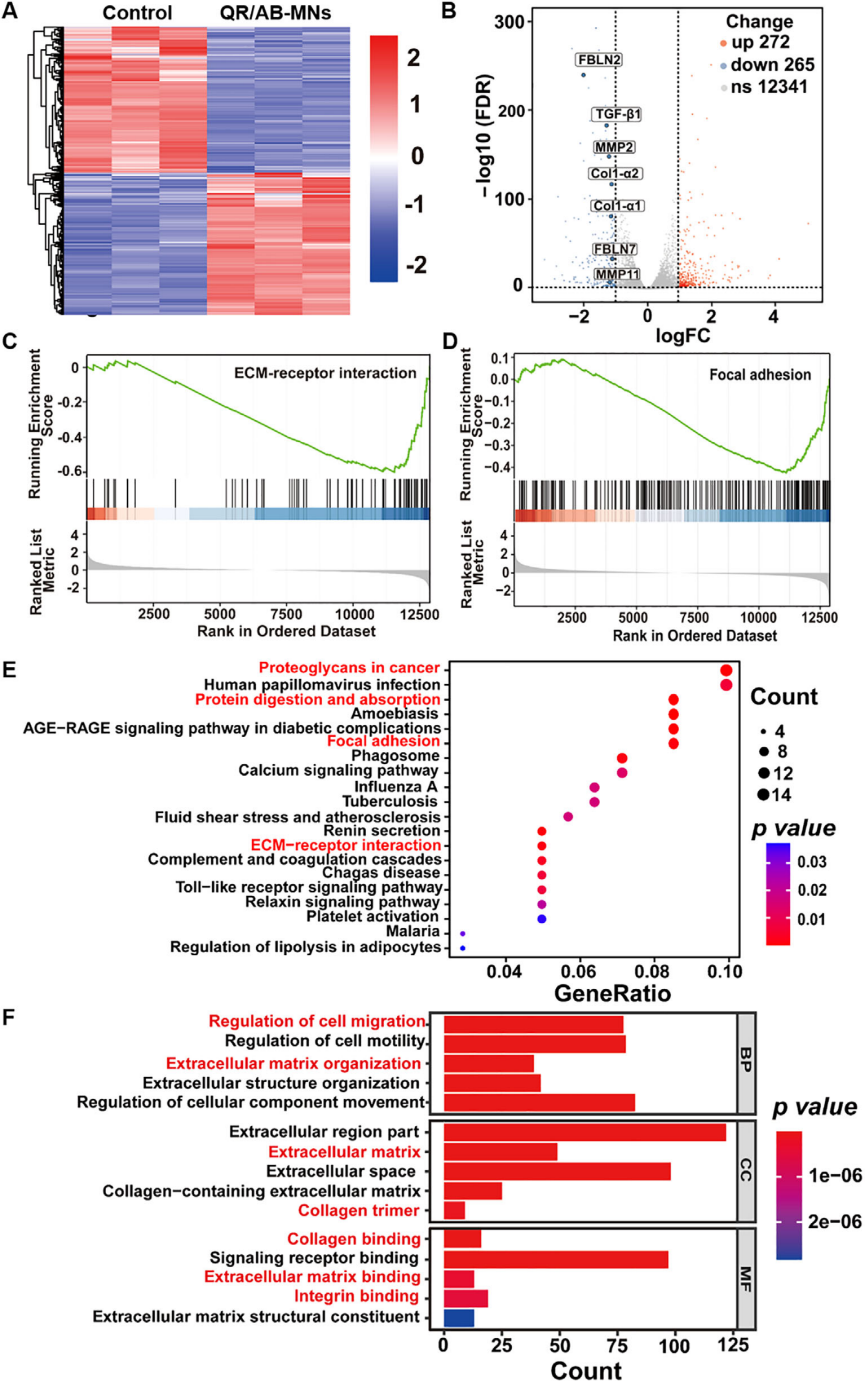
Transcriptomic profiling of HKF cells following QR/AB‐MNs treatment. (A) Heatmap of the transcriptome sequencing results. (B) Volcano plot of differentially expressed genes (DEGs), with red and blue dots indicating significantly up‐ and down‐regulated genes, respectively. (C, D) Gene Set Enrichment Analysis (GSEA) of selected signaling pathways. (E) Kyoto encyclopedia of genes and genomes (KEGG) pathway enrichment analysis with bubble plot. (F) Gene ontology (GO) functional enrichment analysis with bar chart (BP, biological process; CC, cellular component; MF, molecular function.

### 2.5 Evaluation of Burn Wound Healing in Mice In Vivo

The therapeutic efficacy of the core–shell QR/AB‐MNs in promoting the healing of *P. aeruginosa*‐infected deep partial‐thickness to full‐thickness burn wounds was systematically evaluated in a murine model. By day 14 post‐treatment, the QR/AB‐MNs group achieved ∼ 90% wound closure, significantly outperforming the control group (∼ 41%) (Figure 7A,D). Because wound closure in the murine model can be influenced by contraction mediated by the panniculus carnosus, these results were interpreted as evidence of overall wound‐healing efficacy rather than re‐epithelialization alone. Histological analysis at this stage revealed complete, stratified epidermal layers with uniform thickness (Figure 7E), indicative of superior re‐epithelialization. Moreover, the QR/AB‐MNs group exhibited epidermal thickness comparable to that of uninjured skin (Figure 7F and Figure S15), highlighting the role of sustained QR release in promoting regenerative, scar‐sparing repair. Bacterial quantification at days 3, 7, and 10 post‐treatment demonstrated that QR/AB‐MNs rapidly eradicated wound‐associated bacteria, with bacterial burdens progressively declining to near‐sterile levels by day 10 (> 3‐log reduction vs. the control group) (Figure 7B and Figure S16). In contrast, significant bacterial persistence was observed in both the control and QR‐MN groups, underscoring the potent antimicrobial efficacy of AB‐derived degradation products and sustained hydrogen release. To verify the differential release behavior in vivo, FITC and Nile red were incorporated into the SBECD core and PLGA shell, respectively, as surrogate tracers. In a murine burn‐wound model, the core‐associated FITC signal decreased rapidly after MN application, whereas the shell‐associated Nile red signal declined more gradually and remained detectable over 21 days, supporting the temporally differentiated release behavior of the core–shell architecture in vivo (Figure S17).

**FIGURE 7:**
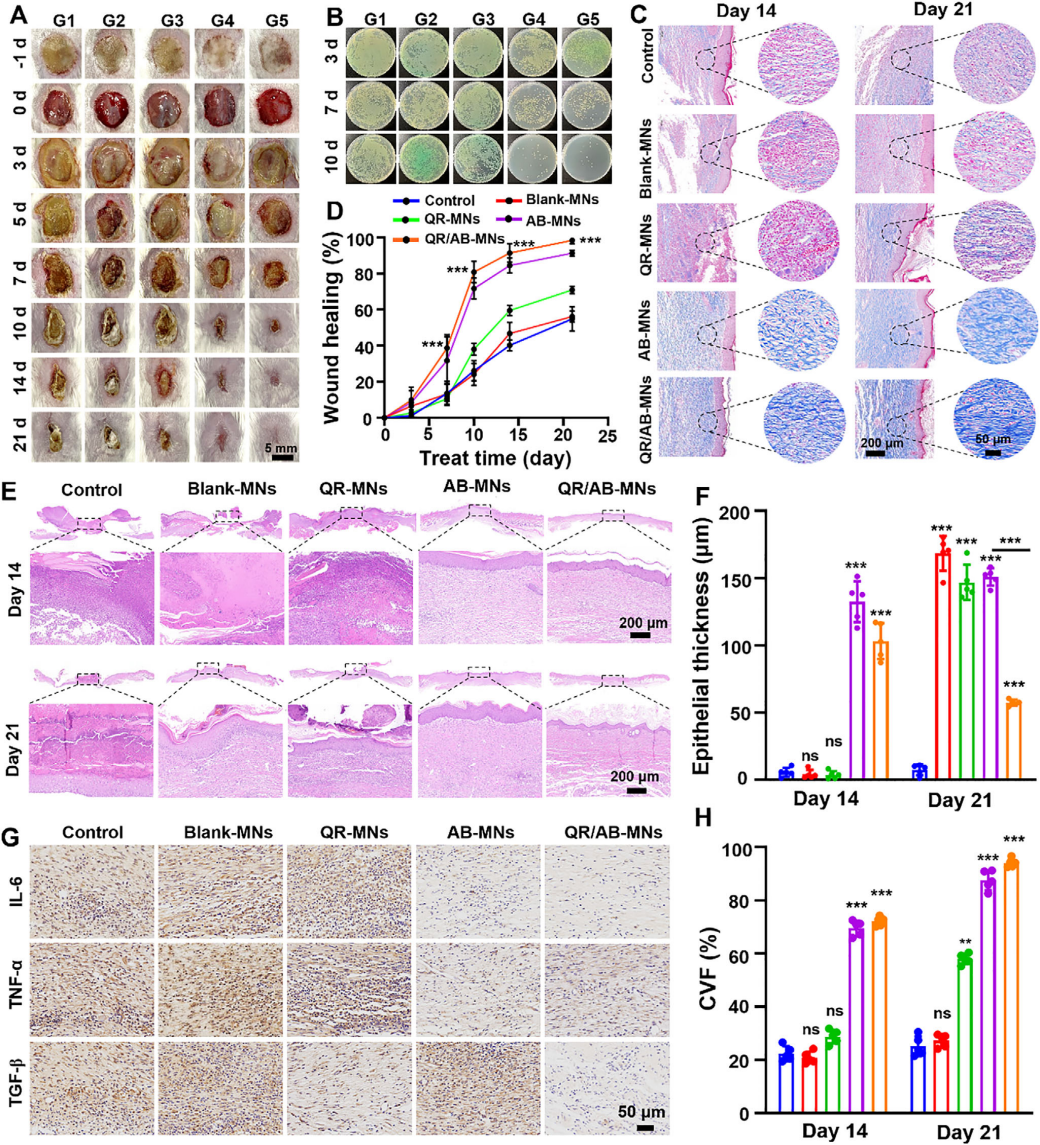
Therapeutic efficacy of QR/AB‐MNs in infected burn wounds in vivo. (A) Representative photographs showing wound healing progression across treatment groups at indicated time points. (B) Bacterial colony formation at wound sites on days 3, 7, and 10 post‐treatment. (C) Representative Masson's trichrome‐stained images of wound tissues on days 14 and 21. (D) Quantitative analysis of wound closure rates among groups. (E) Representative H&E‐stained sections of wound tissues on days 14 and 21 post‐treatment. (F) Statistical analysis of epidermal thickness across treatment groups. (G) Immunohistochemical staining for IL‐6, TNF‐*α*, and TGF‐*β* in wound tissues on day 21. (H) Quantitative analysis of collagen volume fraction among experimental groups. Data are presented as mean ± SD (*n* = 5). ^**^ *p* < 0.01, ^***^ *p* < 0.001, ns indicates no significance. (G1: Control, G2: Blank‐MNs, G3: QR‐MNs, G4: AB‐MNs, G5: QR/AB‐MNs).

Collagen remodeling, an essential determinant of scar‐free healing [52], was examined via Masson's trichrome staining. The QR/AB‐MNs group displayed markedly enhanced collagen deposition and alignment compared with all other groups (Figure 7C,H). By day 14, collagen in the Control, Blank‐MN, and QR‐MN groups remained sparse and disorganized, whereas AB‐MNs and QR/AB‐MNs produced dense, highly ordered fibrillar networks. By day 21, the collagen architecture in the QR/AB‐MNs group closely resembled that of native, uninjured skin, indicating synergistic regulation of ECM remodeling by sustained H_2_ generation and QR release. Immunohistochemical analyses further elucidated the underlying molecular mechanisms. Treatment with AB‐MNs and QR/AB‐MNs significantly reduced expression of the pro‐inflammatory cytokines IL‐6 and TNF‐*α*, consistent with the anti‐inflammatory activity of continuous H_2_ release. Importantly, QR/AB‐MNs also downregulated the pro‐fibrotic mediator TGF‐*β* at day 21 (Figure 7G), suggesting precise modulation of the fibrotic phase and effective prevention of excessive scar formation.

Finally, H&E staining revealed no apparent pathological abnormalities, including tissue degeneration, inflammatory infiltration, necrosis, or structural disruption, in any of the examined organs compared with the control group (Figure S18), further confirming the favorable in vivo biosafety of the MN patches.

### 2.6 Assessment of Scar‐Reduced Healing in a Rabbit Burn Model In Vivo

The capacity of QR/AB‐MNs to promote scarless burn wound healing was further validated using a rabbit ear hypertrophic scar model (Figure 8A), chosen for its close pathophysiological relevance to human scarring. Rabbits were randomly assigned to five treatment groups (Control, Blank‐MNs, QR‐MNs, AB‐MNs, and QR/AB‐MNs). Macroscopic observation revealed that both AB‐MNs and QR/AB‐MNs markedly accelerated wound closure compared to other treatments. However, by day 30, the AB‐MNs group developed elevated, erythematous hypertrophic scars, whereas the QR/AB‐MNs group exhibited smooth, nearly level wound surfaces (Figure 8B), indicating that sustained QR release plays a pivotal role in mitigating hypertrophic scar formation. Histological analysis corroborated these findings. The AB‐MNs group displayed pronounced epidermal thickening and disordered dermal architecture, characteristic of hypertrophic scarring (Figure 8G). In contrast, the QR/AB‐MNs group showed a thinner, more uniform epidermis with near‐normal dermal organization (Figure 8D,G), corresponding to a significantly reduced scar evaluation index (SEI) (Figure 8C). Although QR/AB‐MNs markedly reduced hypertrophic scar formation and improved collagen organization by day 30, longer‐term studies will be required to fully evaluate scar maturation and sustained tissue remodeling beyond this observation period.

**FIGURE 8:**
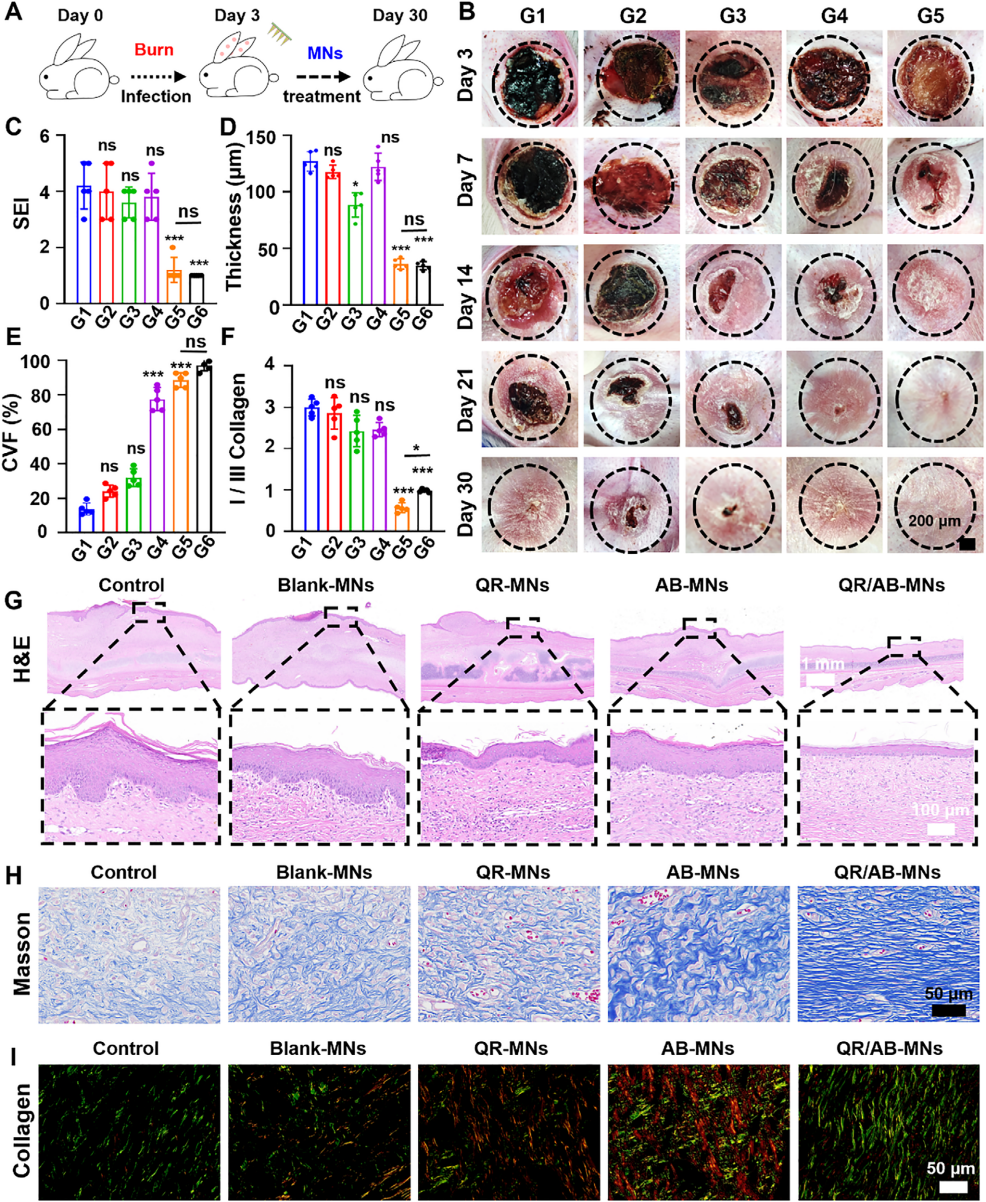
Evaluation of scarless healing induced by QR/AB‐MNs in a rabbit ear burn wound model. (A) Schematic illustration of the application of the QR/AB MNs to the rabbit ear burn wound model. (B) Representative photographs showing wound healing progression in different treatment groups at various time points. (C) Quantitative analysis of the scar elevation index (SEI). (D) Comparative assessment of epidermal thickness among groups. (E) Statistical analysis of collagen volume fraction (CVF) in regenerated tissues. (F) Quantification of type I/III collagen ratio. Representative histological images of healed wounds on day 30 obtained by (G) H&E staining, (H) Masson Trichrome staining, and (I) Sirius Red staining. Data are presented as mean ± SD (*n* = 5). ^*^ *p* < 0.05, ^***^ *p* < 0.001, ns indicates no significance. (G1: Control, G2: Blank‐MNs, G3: QR‐MNs, G4: AB‐MNs, G5: QR/AB‐MNs, G6: Normal).

Masson's trichrome staining further demonstrated dense, well‐organized collagen deposition (blue) in the QR/AB‐MNs group, distinct from the sparse and irregular fibers observed in the control and AB‐MNs groups (Figure 8H). The collagen bundles in QR/AB‐MNs–treated wounds exhibited a parallel, basket‐weave architecture closely resembling that of native skin, accompanied by a higher collagen volume fraction (Figure 8E). Picrosirius red staining under polarized light confirmed these results, revealing balanced collagen maturation (Figure 8I). Notably, the collagen type I/III ratio, which is a key indicator of scar maturity, was markedly reduced from 2.99 ± 0.20 in the control group to 0.57 ± 0.12 in the QR/AB‐MNs group (Figure 8F and Figure S19), supporting attenuation of hypertrophic scar formation and more favorable collagen remodeling following QR/AB‐MN treatment. Immunofluorescence staining of wound tissues further revealed reduced expression of Col‐I, MMP‐2, and TGF‐*β*1 following QR/AB‐MN treatment (Figure S20), consistent with the transcriptomic findings and further supporting the suppression of profibrotic signaling and pathological ECM remodeling in vivo.

Collectively, these findings demonstrate that the synergistic, sustained release of QR and H_2_ from the core–shell QR/AB‐MNs effectively modulates collagen remodeling and fibrotic signaling, thereby achieving regenerative, scar‐sparing healing in severe burn wounds.

## 3 Conclusion

In this study, we developed a core–shell MN patch (QR/AB‐MNs) engineered for synergistic antimicrobial, anti‐inflammatory, and anti‐fibrotic therapy to promote scar‐reduced burn wound healing. The MNs feature a PLGA shell encapsulating QR and an SBECD core loading the H_2_‐releasing prodrug AB, enabling spatially and temporally controlled dual‐drug delivery. The QR/AB‐MNs exhibited potent antibacterial activity, effectively eradicating *P. aeruginosa*, *E. coli*, and *S. aureus*, and disrupting both nascent and mature biofilms through continuous H_2_ generation and boric acid production. In vitro assays demonstrated that sustained H_2_ release significantly reduced intracellular ROS, promoted keratinocyte and fibroblast migration, enhanced angiogenesis, and induced macrophage polarization toward an anti‐inflammatory (M2) phenotype. Simultaneously, QR release suppressed fibroblast hyperproliferation and collagen overproduction by downregulating TGF‐*β* and Col‐1 expression, thereby inhibiting fibrotic remodeling. In in vivo murine burn models, QR/AB‐MNs accelerated wound closure, reduced bacterial burden, attenuated inflammation, and facilitated the regeneration of hair follicles and skin appendages. Further evaluation in a rabbit hypertrophic scar model demonstrated that QR/AB‐MNs achieved scar‐reduced regenerative healing, characterized by highly organized collagen alignment and a collagen type I/III ratio approaching that of normal skin. Collectively, these results highlight QR/AB‐MNs as a multifunctional therapeutic platform that integrates infection control, oxidative stress modulation, and fibrosis suppression to achieve regenerative, scar‐sparing healing. This work establishes a versatile MN‐based strategy for the intelligent, staged delivery of bioactive agents, offering broad potential for the treatment of complex infected and fibrotic wounds.

## 4 Materials and Methods

### 4.1 Materials

PLGA (50:50), AB, H_2_O_2_, and tungsten trioxide (WO_3_) were purchased from Aladdin (Shanghai, China). SBECD and QR were obtained from Macklin (Shanghai, China). Lipopolysaccharide (LPS), Tween 20, and Hoechst staining solution were purchased from Sigma (Missouri, USA). LB nutrient broth, LB nutrient agar, and TSB liquid medium were acquired from Qingdao Hopebiol Technology (Shandong, China). The SYTO‐9/PI kit was sourced from Thermo Fisher (Massachusetts, USA). Crystal violet, calcein‐AM, BCA protein assay kit, EdU staining kit, Live/Dead Cell Staining kit, 2′,7′‐dichlorodihydrofluorescein diacetate (DCFH‐DA), and CCK‐8 kit were purchased from Beyotime Biotechnology Co., Ltd (Shanghai, China). PCNA polyclonal antibody, TGF‐β polyclonal antibody, and Collagen Type I monoclonal antibody were procured from Proteintech Biotechnology (Wuhan, China). Collagen gel was obtained from Hangzhou Shengyou Biotechnology Co., Ltd (Hangzhou, China). *E. coli* (ATCC 25922), *S. aureus* (ATCC 6538), and *P. aeruginosa* (ATCC 9027) were obtained from the China General Microbiological Culture Collection Center (CGMCC), Beijing, China. The HaCaT cell, human umbilical vein endothelial cell line (HUVEC), and murine monocyte‐macrophage cell line (RAW 264.7) were purchased from Procell Life Science & Technology Co., Ltd. (Wuhan, China). HDF and HKF were acquired from Immocell Biotechnology Co., Ltd. (Xiamen, China).

### 4.2 Methods

#### 4.2.1 Fabrication and Characterization of QR/AB‐MN Patches

QR/AB‐MN patches were fabricated using a stepwise centrifugation method. For the shell component, QR was loaded into a PLGA matrix by dissolving it in a 10% PLGA solution. A small aliquot of this solution was dispensed onto the MN mold surface, centrifuged at 4200 rpm for 3 min, and subsequently vacuum‐dried at 40°C overnight. The core component utilized SBECD to load AB. A 100 µL mixture of SBECD and AB solution was added to the dried shell mold, centrifuged at 4200 rpm for 10 min, and vacuum‐dried at 37°C to form the core. The lid structure was created by coating 10 µL of PLGA matrix solution onto the shell surface, followed by centrifugation for 2 min. Thereafter, 100 µL of blank SBECD solution was added and cured under a −0.1 MPa vacuum for 3 h to form the MN backing layer. The final mold was vacuum‐dried at 25°C with 5% relative humidity for 72 h before demolding. Bright‐field and fluorescence images of the MNs were acquired using a stereomicroscope (Leica M165FC) and CLSM (Leica TCS SP8). The microstructure of the MN patches was examined using scanning electron microscopy (SEM, Zeiss GeminiSEM 500). Mechanical properties were characterized with a mechanical tester (Mark ESM303), and individual MN strength was determined from the corresponding force‐displacement curves.

### 4.3 Drug Release Behavior of QR/AB MNs In Vitro

The in vitro release profiles of QR and H_2_ from QR/AB‐MNs were evaluated using UV–vis spectrophotometry (Shimadzu UV‐1240) and hydrogen detection techniques, respectively. For QR release analysis, a calibration curve was constructed at 375 nm using QR standard solutions (1–32 µg/mL). QR‐loaded MNs patches were immersed in PBS containing 2% (v/v) Tween‐20 at 37°C. At predetermined intervals, 0.6 mL of supernatant was collected and replaced with an equal volume of fresh medium to maintain sink conditions. Cumulative QR release was quantified based on UV–vis absorbance measurements. For H_2_ release, preliminary confirmation of hydrogen generation was conducted using WO_3_ colorimetry. MNs incubated in PBS (pH 5.6, 37°C) induced characteristic chromogenic changes in WO_3_ powder. Quantitative hydrogen analysis was performed using a hydrogen microsensor (Unisense FX‐30). MN‐containing vials were sealed and incubated at 37°C under gentle agitation, and H_2_ concentration in the headspace was continuously monitored to determine release kinetics.

### 4.4 Biosafety Assessment

In vitro cytotoxicity and erythrocyte hemolysis assays were conducted to evaluate the biocompatibility of the QR/AB‐MN patches. Immortalized HaCaT at the logarithmic growth phase were co‐cultured with MNs patches, and cell viability was assessed using a LIVE/DEAD cell staining kit. Viable cells exhibited green fluorescence (calcein‐AM), whereas nonviable cells displayed red fluorescence (propidium iodide, PI). For hemolysis analysis, rat erythrocytes were incubated with MN patches for predetermined durations. After centrifugation, the absorbance of the supernatant was measured at 540 nm using UV–vis spectrophotometry, and the hemolysis rate was calculated to quantify erythrocyte damage.

### 4.5 Minimum Inhibitory Concentration

The antibacterial efficacy of compound AB was evaluated by determining its MIC. Serial dilutions of AB were prepared in Luria–Bertani (LB) broth and inoculated with *E. coli*, *S. aureus*, or *P. aeruginosa*. The cultures were incubated at 37°C for 24 h. The MIC was defined as the lowest concentration of AB at which no visible turbidity (i.e., clear broth) was observed, indicating complete inhibition of bacterial growth.

### 4.6 Antibacterial Activity of QR/AB‐MNs In Vitro

The antibacterial activity of QR/AB‐MNs was assessed via a Live/Dead bacterial staining assay. UV‐sterilized QR/AB‐MN patches were immersed in PBS for 5 days to obtain extraction solutions. These extracts were then incubated with *S. aureus*, *E. coli*, or *P. aeruginosa* in LB broth at 37°C for 24 h. Subsequently, 20 µL of each bacterial suspension was plated on LB agar and cultured at 37°C for 12 h to assess bacterial growth. For fluorescence staining, bacterial cells were washed with PBS, treated with QR/AB‐MN extracts at 37°C for 6 h, and stained with SYTO‐9 and PI for 30 min in the dark. After PBS washing, live (green) and dead (red) bacteria were visualized using CLSM. Morphological changes in bacteria following MN treatment were further examined by field‐emission scanning electron microscopy (FE‐SEM).

### 4.7 Evaluation of Antibiofilm Activity

The antibiofilm activity of MNs was evaluated by assessing their ability to inhibit and disrupt *P. aeruginosa* biofilms. For biofilm formation inhibition, *P. aeruginosa* suspensions (2 mL) were added to 6‐well plates containing glass coverslips, followed by the addition of MN extracts. After incubation at 37°C for 48 h, the plates were gently washed three times with PBS and stained with 1 mL of 0.1% (w/v) crystal violet (CV) solution for 15 min. In parallel, biofilms subjected to different treatments were stained with SYTO‐9 and imaged using CLSM. For morphological observation, biofilms were fixed with 4% (w/v) paraformaldehyde for 15 min, dehydrated through a graded ethanol series (50%, 70%, 90%, and 100%), and examined by SEM after gold sputter‐coating. To assess the effect of MN patches on mature biofilms, *P. aeruginosa* (2 mL) was first cultured in 6‐well plates with glass coverslips at 37°C for 48 h to establish mature biofilms. The supernatant was then gently aspirated, and fresh medium containing various MN formulations was added for co‐incubation for 24 h. Biofilm disruption was subsequently evaluated by CV staining, Live/Dead bacterial staining, and SEM analysis.

### 4.8 Cell Proliferation and Migration Assays

Scratch wound healing and Transwell migration assays were performed to evaluate the effects of QR/AB‐MNs on the proliferation and migration of HDFs and HaCaT keratinocytes. Cells were seeded in 6‐well plates and cultured for 24 h to form confluent monolayers. A uniform scratch was then generated across each monolayer using a sterile pipette tip. Dulbecco's modified Eagle medium (DMEM) containing extracts from different MN formulations was added, and cells were incubated for an additional 24 h. Scratch closure was monitored at predetermined time points using an inverted fluorescence microscope (Olympus IX73). The wound‐healing rate was quantified by measuring the scratch width with ImageJ software.

For the Transwell assay, HDFs were suspended in DMEM supplemented with 1% fetal bovine serum (FBS). A 100 µL aliquot of the cell suspension was added to the upper chamber of a Transwell insert, while the lower chamber was filled with DMEM containing 10% FBS and MN extracts. After incubation for 12 h, non‐migratory cells on the upper membrane surface were removed with a cotton swab. Migrated cells on the lower surface were fixed, stained with crystal violet solution, and visualized under an inverted fluorescence microscope. The stained cells were subsequently eluted with 2% acetic acid, and migration was quantified by measuring the absorbance of the eluate using UV–vis spectrophotometry.

### 4.9 Keratinocyte Epithelialization Assay

The keratinocyte epithelialization assay was performed according to previously described methods [27, 53]. Briefly, keratinocytes were seeded into 12‐well plates and cultured at 37°C in 5% CO_2_ until cell attachment was achieved. MN extracts were then added to each well, followed by co‐incubation of the HaCaT cells with the extracts for 24 h. Subsequently, the cells were fixed with 4% paraformaldehyde and stained with 1% crystal violet solution for 15 min. The area of cell colony formation was observed and quantified using an inverted fluorescence microscope.

### 4.10 Assessment of Intracellular ROS Levels in Cells

Intracellular ROS levels were assessed using the DCFH‐DA assay kit. Briefly, HaCaT and HDF cells were seeded into 6‐well plates at a density of 30 000 cells per well and cultured at 37°C under 5% CO_2_ until attachment was achieved. The cells were then co‐incubated with hydrogen peroxide solution (final concentration: 100 µm) for 12 h. Subsequently, cells were treated with different MN extracts for an additional 24 h. Following treatment, the supernatant was aspirated, and the cells were washed three times with PBS. DCFH‐DA solution was then added to the wells, and the cells were incubated at 37°C for 20 min. Intracellular green fluorescence intensity, indicative of ROS levels, was detected and imaged using CLSM.

### 4.11 Macrophage Polarization Assay

The anti‐inflammatory properties of QR/AB‐MNs were evaluated using an in vitro model of M1‐polarized macrophages induced by lipopolysaccharide (LPS). Briefly, RAW 264.7 cells were seeded into 6‐well plates and polarized toward the M1 phenotype by incubation in medium containing 10 ng/mL LPS for 24 h. Cells were subsequently treated with MN extracts for an additional 24 h. Following treatment, cells were harvested and sequentially stained for surface markers (APC‐conjugated anti‐CD86 antibody to identify M1 macrophages) and intracellular markers (PE‐conjugated anti‐CD206 antibody to identify M2 macrophages). The proportions of cells exhibiting the M1 (CD86^+^) and M2 (CD206^+^) phenotypes were then determined by flow cytometry (Beckman CytoFLEX).

### 4.12 EdU Staining Assay

Cell proliferation in scar‐derived fibroblasts (HKF cells) was assessed using the BeyoClick EdU‐488 Kit. Briefly, HKF cells were co‐cultured with 14‐day MN extracts for 24 h. Cells were then incubated with Click‐iT EdU reaction cocktail for 2 h. Following incubation, cells were fixed with 4% paraformaldehyde for 15 min at room temperature. After fixation, cells were incubated with Click‐iT reaction buffer for 30 min at room temperature in the dark. Finally, nuclei were counterstained with Hoechst 33342 for 5 min. EdU incorporation signals (green fluorescence) were visualized and imaged using an inverted fluorescence microscope.

### 4.13 Scar Fibroblast Colony Formation Assay

Colony formation by HKF was assessed using a monolayer culture method. HKFs were seeded into 6‐well plates at a density of 500 cells per well. Cells were then treated with 14‐day MN extracts. After 14 days of culture, viable cell colonies were fixed with 4% paraformaldehyde for 15 min and stained with 1% crystal violet solution for 5 min.

### 4.14 Collagen Gel Contraction Assay

The collagen gel contraction assay was performed as previously described [54]. Briefly, HKF cells were uniformly mixed with 500 µL of collagen gel solution. The mixture was then dispensed into 24‐well plates. Plates were incubated at 37°C for 30 min to allow collagen polymerization. Following polymerization, 14‐day MN extracts were added to each well, and the gels were cultured at 37°C under 5% CO_2_ for 48 h. Finally, gels were carefully detached from the wells using a collagen gel releaser. The contracted gel area was photographed under a microscope for each group.

### 4.15 Western Blot Analysis

Western blotting was performed to evaluate the expression levels of Col‐1, TGF‐β, and PCNA in QR/AB MN‐treated HKF cells. Following treatment, HKF cells were lysed using RIPA buffer. Total protein concentration was determined using a BCA assay kit. Proteins were separated by SDS‐PAGE on polyacrylamide gels and transferred to membranes. Membranes were immunoblotted with the following primary antibodies: anti‐Col‐1 (1: 3000), anti‐TGF‐β (1: 5000), and anti‐PCNA (1: 5000). Protein bands were visualized using an appropriate secondary antibody and chemiluminescence detection system.

### 4.16 Therapeutic Efficacy of QR/AB‐MNs in A Murine Burn Wound Model In Vivo

Male BALB/c mice (6–8 weeks) were purchased from Shanghai SLAC Laboratory Animal Co., Ltd. All animal experiments were conducted in accordance with the ARRIVE 2.0 guidelines and were approved by the Institutional Animal Care and Use Committee of Wuhan University (WP20240111). Mice were anesthetized by intraperitoneal injection of 2% sodium pentobarbital at a dose of 50 mg/kg, followed by removal of the dorsal hair using depilatory cream. Animals were randomly allocated to treatment groups using a computer‐generated randomization sequence, and investigators performing wound‐area quantification, bacterial burden analysis, and histological assessment were blinded to group allocation. Mice were anesthetized via intraperitoneal injection of 2% sodium amobarbital, and dorsal hair was removed using depilatory cream. Deep partial‐thickness to full‐thickness burns (approximating deep third‐degree burns) were created on the dorsal skin using a calibrated burn device (120°C) applied for 10 s. At 24 h post‐burn, necrotic tissue was debrided with sterile surgical scissors, and wounds were inoculated with 100 µL of *P. aeruginosa* suspension (10^7^ CFU/mL). Successfully modeled mice were randomly assigned to five treatment groups (*n* = 5): Control (untreated), Blank MNs (Blank‐MNs), QR‐loaded MNs (QR‐MNs), AB‐loaded MNs (AB‐MNs), and QR/AB co‐loaded MNs (QR/AB‐MNs). MN patches were gently applied to the wound site for 10 min, after which the backing layer was removed. Wound progression was monitored periodically, and wound areas were photographed and documented on days 0, 3, 7, 10, 14, and 21. Wound exudates were collected on days 3, 7, and 10 using sterile cotton swabs for bacterial load quantification. At specified endpoints, wound tissue samples were harvested, fixed in 4% paraformaldehyde, and processed for hematoxylin and eosin (H&E) staining, Masson's trichrome staining, and immunohistochemical (IHC) analysis.

### 4.17 Assessment of MN Efficacy in Reducing Scar Formation in a Rabbit Burn Model In Vivo

New Zealand White male rabbits (3–4 months) were anesthetized by intravenous injection of pentobarbital sodium (30 mg/kg). Three full‐thickness (third‐degree) burn wounds were created on each ear using a calibrated burn device, and the perichondrium was completely excised using a scalpel. Each wound was subsequently inoculated with 200 µL of a *P. aeruginosa* suspension (10^9^ CFU/mL). After bacterial inoculation for 72 h, the wounds were randomly assigned to the Control, Blank‐MNs, QR‐MNs, AB‐MNs, or QR/AB‐MNs groups. Wound sites were photographed throughout the treatment period to monitor healing. On day 30 post‐burn, scar tissue was harvested, fixed in 4% paraformaldehyde, and processed for histological evaluation using H&E staining, Masson's trichrome staining, and Picrosirius red staining. The SEI was calculated as the ratio of scar dermal thickness to that of adjacent uninjured skin, and the collagen organization was assessed by comparison with uninjured rabbit‐ear skin processed in parallel [55]. All histological sections were examined using an inverted fluorescence microscope. Prespecified inclusion and exclusion criteria were applied, and the experiments were conducted in accordance with the ARRIVE 2.0 guidelines.

### 4.18 Statistical Analysis

Statistical analysis was performed with the aid of GraphPad Prism software, Angio Tool software, and ImageJ software. All data were expressed as mean ± standard deviation (SD), and at least 3 samples per experiment were used for statistical analysis of the data. Statistical analyses were performed using ANOVA and Tukey's multiple comparison test, and *p* < 0.05 was considered statistically significant (^*^ *p* < 0.05, ^**^ *p* < 0.01, ^***^ *p* < 0.001, ^****^ *p* < 0.0001).

## Author Contributions

**Yongnian Zeng**: conceptualization, methodology, data curation, investigation, writing – original draft. **Zhigang Chu**: methodology, data curation, validation, writing – original draft. **Huiyuan Yang**: methodology, validation, investigation, data curation. **Wen Zhang**: methodology, investigation. **Peng Jiang**: methodology, investigation. **Jiapeng Lei**: methodology, data curation. **Siyu Zhang**: methodology, data curation. **Qiongfang Ruan**: methodology. **Shuhua Liu**: conceptualization, supervision, writing – review and editing, validation, funding acquisition, project administration. **Wei Li**: conceptualization, validation, supervision, project administration, funding acquisition, writing – review and editing.

## Conflicts of Interest

The authors declare no conflicts of interest.

## Supporting information




**Supporting File**: advs78512‐sup‐0001‐SuppMat.docx.

## Data Availability

The data that support the findings of this study are available from the corresponding author upon reasonable request.
