# Supplementary material for: Phase‐Programmed Microneedles Enable Sequential Hydrogen and Quercetin Release for Scar‐Reduced Regeneration of Burn Wounds
Source: Adv Sci (Weinh). 2026 Oct 28:e78512. Online ahead of print. doi: 10.1002/advs.78512 (PMC13715928; doi:10.1002/advs.78512)
Supplement: Supplementary file 1 [file ADVS-9999-e78512-s001.docx]

**Supporting Information**

**Figure S1. (**A) Schematic illustration of the fabrication process of the QR/AB-MN patch. (B) Representative images of the fabricated QR/AB-MN patch.

**Figure S2.** Representative bright-field and fluorescence images of the core-shell MNs with (A) or without (B) lid. Nile red stains the MNs shell and lid (red), and rhodamine green stains the MNs core (green). Representative bright-field and fluorescence images of the MNs with (C) or without (D) lid after insertion into simulated skin, showing the drug release from the core at different time points.

**Figure S3. (**A) Elemental mapping of C, S, and Na distributions in the longitudinal section of a single QR/AB-MN. (B) Optical microscopy images showing vertical (upper) and cross-sectional (lower) views of the core-shell MNs structure.

**Figure S4**. H&E-stained section of burn-eschar-covered porcine skin following penetration with core-shell MNs.

**Figure S5**. Standard curves for quantifying the drug loading capacity of QR (A) and AB (B). (C) Standard curve for assessing QR concentration during in vitro release from the MNs shell. (D) Temporal fluorescence images of core-shell MNs with lid following insertion into simulated skin.

**Figure S6. (**A) Representative SEM images of QR/AB-MNs after immersion in simulated body fluid (SBF) for varying durations. (B) SEM images illustrating the surface morphological changes of the QR/AB-MNs shell following exposure to SBF over time.

**Figure S7.** (A) Representative images of erythrocytes after co-culture with MNs from different groups. (B) Statistical analysis of hemolysis rates across different groups. (C) Cytotoxicity of QR/AB-MNs to three kinds of skin cells (HaCaT, HDF, and HUVEC), respectively.

**Figure S8.** (A) Representative images of *E. coli*, *S. aureus*, and *P. aeruginosa* after 24-hour co-culture with varying concentrations of AB. (B) Bacterial colony formation on agar plates following 24-hour co-culture with QR/AB-MNs. (C) Mature biofilm disruption assessment: (a) crystal violet-stained mature biofilms, (b) confocal 3D reconstructions of SYTO-9-stained biofilms post-treatment, (c) SEM images of gradient-dehydrated mature biofilms after different treatments. (D) Statistical analysis of mature biofilm disruption rates across groups. Data represent mean ± SD (n = 5).

**Figure S9**. Antimicrobial activity of AB byproducts (A) and H_2_ (B) at different concentrations against representative bacterial strains. Quantitative analysis of *E. coli* (C), *S. aureus* (D), and *P. aeruginosa* (E) growth following treatment with AB byproducts at different concentrations. Blue dashed boxes indicate treatment conditions under which bacterial growth was significantly inhibited. Data are presented as mean ± SD (n = 3). ****P* < 0.001, ns indicates no significance.

**Figure S10**. In vitro antibacterial and antibiofilm activities of different MN formulations. (A) Representative fluorescence images of *E. coli* and *S. aureus* after treatment with Control, Blank-MNs, QR-MNs, AB-MNs, or QR/AB-MNs. Live and dead bacteria are shown in green and red, respectively. (B, D) Viable bacterial counts of *E. coli* (B) and *S. aureus* (D), determined by the colony-counting method. (C) Representative images of crystal violet-stained *E. coli* and *S. aureus* biofilms following the indicated treatments. (E, F) Bacterial survival rates of *E. coli* (E) and *S. aureus* (F). (G, H) Quantification of *E. coli* (G) and *S. aureus* (H) biofilm biomass by measuring the absorbance of crystal violet at 570 nm. (I) Viable counts of *P. aeruginosa* following the indicated treatments. Data represent mean ± SD (n = 3). ****P* < 0.001, ns indicates no significance.

**Figure S11.** (A) Representative images from scratch wound assays demonstrating HaCaT cell migration following treatment with different MNs. (B) Quantitative analysis of scratch closure rates. (C) Bright-field images documenting HDF cell migration after treatment with various MNs. (D) Statistical evaluation of HDF cell migration capacity across experimental groups. All data represent mean ± SD (n = 5).

**Figure S12.** Flow contour images of LPS-induced M1/M2 levels in RAW 264.7 cells.

**Figure S13**. Selective effects of QR on normal human dermal fibroblasts (HDFs) and human keloid fibroblasts (HKFs). Cell viability of HDFs (A) and HKFs (B) following treatment with QR at the indicated concentrations under matched experimental conditions. Data are presented as mean ± SD (n = 3). ****P* < 0.001, ns indicates no significance.

**Figure S14.** Relative expression levels of Col-Ⅰ (A), TGF-β (B), and PCNA (C) proteins in each group. Data are presented as mean ± SD (n = 3). **P* < 0.05, ****P* < 0.001, ns indicates no significance.

**Figure S15**. (A) Representative histological images of normal mouse skin stained with H&E. (B) Statistical analysis comparing the epidermal thickness of mice in each group with that of normal mice at 21 days.

**Figure S16**. Bacterial survival rates in mouse wounds at different time points. Data represent mean ± SD (n = 5). ****P* < 0.001, ns indicates no significance.

**Figure S17.** (A) Representative in vivo fluorescence images obtained by animal imaging system showing drug release from the core and shell parts of MNs at different time points after insertion into burn wounds in mice. (B) Statistical analysis of drug release from the core. (C) Statistical analysis of drug release from the shell. Data are presented as mean ± SD (n = 5). **P* < 0.05, ***P* < 0.01, ****P* < 0.001, ns indicates no significance.

**Figure S18.** Histological evaluation of major organs following treatment with different MN formulations. Representative H&E-stained sections of the heart, liver, spleen, lung, and kidney collected from mice in the Control, Blank-MNs, QR-MNs, AB-MNs, and QR/AB-MNs groups on day 21 after treatment.

**Figure S19**. Representative images of normal rabbit ear skin stained with H&E (A), Masson’s trichrome (B), and Sirius red (C) viewed under polarized light.

**Figure S20.** (A) Representative immunofluorescence images of Col-Ⅰ in wound tissues from each group (blue: DAPI; red: Col-Ⅰ). (B) Statistical analysis of mean fluorescence intensity of Col-Ⅰ among groups. (C) Representative immunofluorescence images of MMP-2 in wound tissues (blue: DAPI; purple: MMP-2). (D) Statistical analysis of mean fluorescence intensity of MMP-2. (E) Representative immunofluorescence images of TGF-β1 in wound tissues (blue: DAPI; green: TGF-β1). (F) Statistical analysis of mean fluorescence intensity of TGF-β1. Data are presented as mean ± SD (n = 5). ***P* < 0.01, ****P* < 0.001, ns indicates no significance.

**Table S1.** Comparison of QR/AB-MNs with representative advanced wound dressing platforms for infected wound treatment.

| **Technique** | **Major advantages** | **Main limitations** | **Ref** |
| --- | --- | --- | --- |
| ROS-responsive hydrogel | (a) Respond to elevated ROS microenvironment of infected wounds.  (b) Favorable biocompatibility and maintain moist wound microenvironment.  (c) Realize local antibacterial and anti‑inflammatory intervention. | (a) Mainly acts on wound surface, limited deep‑tissue penetration into burn bed.  (b) Hard to achieve phase‑sequential release for long‑term anti‑scar therapy.  (c) Potential risk of premature drug leakage under oxidative stimulation. | [1-5] |
| NO-releasing nanofibrous dressing | (a) Broad‑spectrum bactericidal effect against drug‑resistant bacteria.  (b) Porous structure absorbs wound exudate effectively.  (c) Good conformal attachment onto irregular wound surface. | (a) Therapeutic payloads distribute only at wound surface, poor deep tissue penetration.  (b) Difficult to realize sustained and delayed anti‑fibrotic release for scar prevention.  (c) High NO burst release may induce local cytotoxicity. | [6,7] |
| Multifunctional hydrogel MN patch | (a) Minimal invasiveness.  (b) Improved tissue penetration.  (c) high local drug utilization.  (d) hydrogel-mediated wound protection. | (a) Reported mainly in diabetic wound models.  (b) limited evidence for infection control in deep burn wounds and prevention of hypertrophic scarring. | [8-11] |
| Hydrogen-releasing hydrogel dressings | (a) Hydrogen‑mediated antioxidation to alleviate wound oxidative stress.  (b) Good biocompatibility, no obvious cytotoxicity of H_2_.  (c) Easy preparation and large‑scale production potential. | (a) Low solubility of hydrogen leads to transient short‑term bioavailability.  (b) Limited penetration depth into burn wound tissue.  (c) Lacking co‑loaded anti‑fibrotic agents for late‑stage scar suppression. | [12] |
| **Core-shell QR/AB-MNs** | (a) Realizing precise spatiotemporal dual‑phase regulation matching inflammation‑proliferation‑remodeling cascades of burn wounds.  (b) Enhanced penetration depth into burn wound bed.  (c) Early sustained H_2_‑boric acid release for anti‑bacterial and antioxidative therapy.  (d) Delayed long‑term QR release (~30 days) to suppress fibroblast over‑activation and collagen fibrosis.  (e) Integrating anti‑infection, inflammation modulation, and scar-reduced skin regeneration in one platform. | (a) Clinical‑relevant large‑animal experiments and comprehensive biosafety evaluation are required before clinical translation. |  |

**References**

1.	Yi, D., et al. Injectable ROS/pH-responsive hydrogel for synergistic chemo-photodynamic tumor therapy and enhanced wound healing. *Adv Healthc Mater* 15, e02349 (2026).

2.	Zhao, M., et al. Stem cell-derived nanovesicles embedded in dual-layered hydrogel for programmed ros regulation and comprehensive tissue regeneration in burn wound healing. *Adv Mater* 36, e2401369 (2024).

3.	Zhang, H., et al. Hydrogel-based bioactive synthetic skin stimulates regenerative gas signaling and eliminates interfacial pathogens to promote burn wound healing. *ACS Nano* 19, 15002-15017 (2025).

4.	Du, K., et al. Multifunctional bioactive peptide-laden and adhesion-switchable dual-crosslinked hydrogel for accelerated healing of infected deep burn wounds. *Acta Biomater* 215, 21-36 (2026).

5.	Zhang, L., et al. An ultrafast self-gelling versatile hydrogel for rapid infected burn wound repair in military medicine. *Adv Funct Mater* 36, e25541 (2026).

6.	Wang, Y., et al. Janus fibrous dressing with controllable nitric oxide-releasing and unidirectional exudate transport activities for bacteria-infected burn wound therapy. *Adv Funct Mater* 35, 2503992 (2025).

7.	Su, L., et al. Low-temperature trigger nitric oxide nanogenerators for anti-biofilm and wound healing. *Adv Fiber Mater* 6, 512-528 (2024).

8.	Elakkawi, M. M. J., et al. Study on the mechanism of hUCMSCs-derived 3D exosome combined with HAMA microneedles in the treatment of deep second-degree burns. *Bioact Mater* 56, 468-482 (2026).

9.	Jia, Q., et al. Spatiotemporally orchestrated microneedle patch synergizing photodynamic therapy, ros scavenging, and photosynthetic oxygenation for chronically infected wound healing. *Nano Lett* 25, 15594-15604 (2025).

10.	Zhang, G., Wei, M., Zhang, Z., Chen, S. & Wan, W. Multifunctional microneedle patch with antibacterial, antioxidant, and pro-regenerative properties for scarless wound healing. *ACS Nano* 20, 10707-10723 (2026).

11.	Tian, S., et al. Multifunctional hydrogel microneedle patches modulating oxi-inflamm-aging for diabetic wound healing. *Small* 20, e2407340 (2024).

12.	Zhu, Y., et al. Two-dimensional Mg2Si nanosheet-enabled sustained hydrogen generaion for improved repair and regeneration of deeply burned skin. *Adv Healthc Mater* 12, 2201705 (2023).
